# Genome-wide identification and characterization of *ADH* gene family and the expression under different abiotic stresses in tomato (*Solanum lycopersicum* L.)

**DOI:** 10.3389/fgene.2023.1186192

**Published:** 2023-09-01

**Authors:** Qingdong Zhu, Yading Han, Wentao Yang, Hang Zhu, Guangtong Li, Ke Xu, Mingxin Long

**Affiliations:** School of Biological Sciences, Jining Medical University, Rizhao, China

**Keywords:** ADH, gene family, bioinformatics, stress, tomato

## Abstract

The *SlADH* gene plays a key role in environmental stress response. However, limited studies exist regarding the tomato *SlADH* gene. In this study, we identified 35 *SlADH* genes in tomato by genome-wide identification. Among the 12 chromosomes of tomato, *SlADH* gene is distributed on 10 chromosomes, among which the 7th and 10th chromosomes have no family members, while the 11th chromosome has the most members with 8 family members. Members of this gene family are characterized by long coding sequences, few amino acids, and introns that make up a large proportion of the genetic structure of most members of this family. Moreover, the molecular weight of the proteins of the family members was similar, and the basic proteins were mostly, and the overall distribution was relatively close to neutral (pI = 7). This may indicate that proteins in this family have a more conserved function. In addition, a total of four classes of cis-acting elements were detected in all 35 *SlADH* promoter regions, most of which were associated with biotic and abiotic stresses. The results indicate that *SlADH* gene had a certain response to cold stress, salt stress, ABA treatment and PEG stress. This study provides a new candidate gene for improving tomato stress resistance.

## 1 Introduction

Alcohol dehydrogenase (ADH, EC 1.1.1.1), a part of the dehydrogenase superfamily, is a zinc-binding enzyme that catalyzes the mutual conversion between acetaldehyde and ethanol as a dimer, relying on the NAD(P) cofactor (Strommer, 2011). It is a well-studied member of the medium-length dehydrogenase/reductase (MDR) protein superfamily. And is found in a wide range of organisms (Speirs et al., 1998; Koch et al., 2000; Manriquez et al., 2006; Singh et al., 2010; Jin et al., 2016). The *ADH* gene family is a large family, mainly divided into three subfamilies: Short-chain dehydrogenase/Reductase (SDR) -ADH (containing about 250 amino acid residues), medium-chain dehydrogenase/reductase (MDR) -ADH (containing about 350 amino acid residues), and long-chain dehydrogenase/reductase (LDR) -ADH (600–750 amino acid residues or about 385–900 amino acid residues) (Alka et al., 2013). In plants, most *ADHs* contain zinc ligands and belong to the medium-chain protein subfamily (Yeganova et al., 2004). These enzymes are widely involved in metabolic processes and have a positive role in increasing resistance to biotic and abiotic stresses (Linskens and Schrauwen, 1966; Jacobs et al., 1988; Tadege et al., 1999; Garabagi et al., 2005; Bailey-Serres and Voesenek, 2008; Banach et al., 2009; Bolte et al., 2011).

*ADH* is ubiquitously present in both animals and plants, though it is more commonly known in the latter. Plants require respiration at all times to energize their physiological functions. However, when there’s insufficient oxygen for normal respiration, the aerobic process transitions to anaerobic respiration. Plants produce large amounts of alcohol during anaerobic respiration, and alcohol dehydrogenase (ADH) is a key enzyme in the ethanol fermentation pathway and subsequent adaptive anaerobic metabolism of plant tissues (Taneja and Mande, 1999). For example, during submergence stress, many plants alter their structure by elongating their stems to produce aerenchyma, which delivers oxygen from leaves exposed to air to submerged plant parts (Sasidharan and Voesenek, 2015). Concurrently, there’s an unavoidable transition from aerobic to anaerobic glycolysis, termed fermentation. Under these conditions, typical transcription and translation processes halt, and “anaerobic peptides” are preferentially synthesized. Although less energy is produced in this way, it is thought to play an important role in cell survival. Studies have shown that the submergence tolerance of plants is proportional to the change of *ADH* activity in response to submergence. *ADH* gene has been considered as an important candidate gene for genetic manipulation of submergence tolerance by improving the adaptability of plants to hypoxia (Jacobs et al., 1988; Takahashi et al., 2014). Research by Komatsu et al. revealed that soybean GmADH was particularly induced by waterlogging, yet these genes remained relatively stable under cold and drought stresses (Komatsu et al., 2010; Komatsu et al., 2012). In addition, *ADH* family genes have also been found to be involved in other abiotic stresses such as cold and drought. Studies have shown that in cereal crops and Arabidopsis, the *ADH* gene is a chilling-induced gene and plays a very important role in plant resistance to chilling stress (Kato-Noguchi and Yasuda, 2007; Zhang et al., 2023). The *ADH* gene is involved in the cold resistance of forest strawberry (*Fragariavesca*) and is a good molecular marker candidate for cold stress. When maize seedlings were exposed to low temperature stress, the activity of *ADH* was also found to increase. Conversely, in the *ADH1*-*ADH2* double deletion mutant, membrane lipid peroxidation intensified, leading to pronounced cellular damage (Gao et al., 2022).

*ADH* also plays a vital role in regulating fruit flavor. The expression of the *ADH* gene is stringently regulated in mature fruits. Owing to ADH’s function in alcohol production, diminished ADH activity in fruits can result in a subdued and modified flavor profile (Jelski et al., 2014). Mature tomato fruit has a high content of ADH2, but does not contain ADH1. In transgenic tomato fruits with *ADH2* overexpression, there was a notable rise in C6 alcohol levels compared to C6 aldehydes, leading to a richer tomato flavor. This enhancement was especially linked to elevated concentrations of Z-3-hexenol (Speirs et al., 1998). Similarly, in grapes with a small *ADH* gene family, fruits produce higher levels of ADH and exhibit increased ADH activity at late maturation (Yamauchi et al., 1990).

Tomato stands out as one of the paramount vegetable crops worldwide, serving as a crucial model crop, the tomato frequently features in plant science research. It has always been favored in the international and domestic markets, and is one of the horticultural crops with the highest economic benefits in the world. In recent years, people’s demand for tomato is increasing day by day, and the demand for planting area is also gradually increasing, but the resulting planting problems are also increasing significantly. These consequent problems caused a large reduction in tomato production, resulting in great economic losses. To counteract severe biotic and abiotic stresses, plants have naturally evolved a repertoire of defensive strategies, allowing them to swiftly adapt to their intricate surroundings, mitigate harm, and compete for vital resources (Atkinson and Urwin, 2012). Faced with complex environmental conditions, plants have a complex and comprehensive response, including the *ADH* gene. At the same time, as a characteristic of tomato fruit, the flavor of tomato is also regulated by *ADH* gene, so it is necessary to study the *ADH* gene family in depth. At present, there are few studies on tomato *ADH* gene family, and the specific contribution of each family to tomato stress resistance and flavor is relatively vague. Studying tomato *SlADH* gene can provide important information for tomato molecular breeding. In this study, we delved into the gene structure, chromosome location, phylogenetic tree and transcriptome-based expression data of tomato *SlADH* gene family, and constructed the expression profiles of this gene in different tissues under different biotic and abiotic stresses. In this study, the *ADH* gene family was analyzed in detail, which could provide a reference for the study of the role of *ADH* gene family in exploring the response of tomato to biotic and abiotic stress.

## 2 Materials and methods

### 2.1 Plant materials and treatments

Tomato cv. MoneyMaker was preserved and propagated in our laboratory. The setting of the plant growth chamber was as follows: with a photoperiod of 16 h/8 h (day/night), 22°C/18°C (day/night) with a humidity of 60%/50% (day/night). The leaves were collected when tomato seedlings grew to the five-leave stage after treatment at 0, 6, 12, 24, and 48 h, respectively.

For the cold stress treatment, the temperature of the chamber was set at 4°C. For the drought stress treatment, tomato plants were subjected to a 10% polyethylene glycol (PEG) solution. For the salinity stress treatment, tomato plants were subjected to a 200 mM NaCl solution. For the ABA treatment, a 0.1 mM ABA solution was evenly sprayed on the surface of tomato seedlings until droplets began to form at the edges of the leaves.

### 2.2 Identification and sequence analysis of *SlADH* genes

Tomato genome sequence version SL4.0 and the genome annotation file version ITAG4.0 were downloaded from the Phytozome database (https://phytozome.jgi.doe.gov/pz/portal.html). The seed alignment files of the *ADH* domain (PF00107: ADH_zinc_N and PF08240: ADH_N) were downloaded from the Pfam (http://www.ebi.ac.uk/interpro/entry/pfam/#table) database. HMMER3 software was used to search the protein containing the ADH_N and ADH_zinc_N (Altermann et al., 1999), with an e-value cutoff of 0.01 (Finn et al., 2011), then all the candidate protein sequences were submitted to Pfam, CDD and SMART databases to verify the domains. Only a protein containing two domains will be considered an ADH protein.

### 2.3 Chromosome location analysis of tomato *SlADH* genes

The chromosomal position data of *SlADH* genes were extracted from the genome annotation file. The figure showing the location of *SlADH* genes were generated with the Show Genes on Chromosome model of TBtools software (Chen et al., 2020).

### 2.4 Phylogenetic tree construction of SlADH proteins in tomato

The ADH proteins of the other species were downloaded from the NCBI database (Onodera et al., 2023), and the multiple sequence alignment was performed using MUSCLE with the default options (Kumar et al., 2016). The phylogenetic tree was constructed using the Neighbor-joining (NJ) method with 1,000 bootstraps.

### 2.5 Analysis of conserved motifs and gene structure of *SlADH* genes

The MEME software was used to identify the conserved motifs among tomato *ADH* genes with the common set value (Yum et al., 1991), the length of motifs were range 6–20, the maximum number of motifs was 10. The gene structure information of *SlADH* genes were obtained from the genome annotation file (version: ITAG4.0), containing the CDS (coding sequence) region, the UTR (untranslated region) region and the intron region.

### 2.6 Analysis of cis-acting elements of tomato *SlADH* genes

The 1.5 kb upstream sequence of *SlADH* genes were obtained from the genome sequence (version: SL4.0) by perl script. Meanwhile, PlantCARE (https://bioinformatics.psb.ugent.be/webtools/plantcare/html/) database was used to predict the cis-elements of the promoter regions of *SlADH* genes, and a Python script was used to stat the cis-elements data, a heatmap was used to show the distribution of cis-elements.

### 2.7 Synteny analysis of *SlADH* genes

The gene duplication events were analyzed by the One step MCScanX model of Comparative Genomics function of TBtools, and the Advanced Circos model was used to show the duplication events of *SlADH* genes in tomato.

In addition, the genome sequence file and the genome annotation file of *A. thaliana*, *Solanum tuberosum* and *Zea mays* were downloaded from Phytozome database, then the OneStepMCScanX model of TBtools was used to analyze the synteny relationship of orthologous *ADH* genes between the tomato and *A. thaliana*, *S. tuberosum, Z. mays*.

### 2.8 Transcriptome expression pattern of *SlADH* genes

The transcriptome raw data with accession: PRJNA634438 was downloaded from the NCBI database, containing drought stress treatment and heat stress treatment were used in this study. The fastp (v0.23.3) software was used to filter the RNA-seq data (Chen et al., 2018), the hisat2 software was used to map the raw data to the tomato genome sequence (Kim et al., 2019), the featurecounts of Rsubread package in R was used to the genes quantification (Mo et al., 2021). The heatmap model of TBtools was used to display the heatmap of *SlADH* gene expression.

### 2.9 Quantitative real-time PCR analysis of *SlADH* genes

After stress treatment on tomato plants, we selected 16 genes for q-PCR to analyze the response of *SlADH* family members to stress. Primer5 (Supplementary Table S1) was used for primer design. Primers were ordered from Sogon Bioscience Co., Ltd., and the dye used for qRT-PCR was purchased from Biorun (BioRun Biosciences Co., Ltd., Wuhan, China). The system of qRT-PCR is referred to Mo’s study (Mo et al., 2021).

### 2.10 Statistical analyses

All the data are presented as the means ± standard deviations (SDs). Statistical analysis was carried out with one-way analysis of variance (ANOVA) in SPSS software (SPSS, Chicago, United States). For all comparisons, *p* < 0.05 was considered statistically significant, represented in the figures by asterisks. All experiments were repeated three times.

## 3 Results

### 3.1 SlADH gene identification and chromosomal distribution analysis

A total of 35 *SlADH* genes were identified in tomato genome (Table 1). The SlADH gene lengths varied, ranging from 892 bp (*SlADH17*) to 14,237 bp (*SlADH8*). Only two of the *SlADH* genes had genome lengths less than 2,000 bp (*SlADH17*, *SlADH28*). The number of amino acids varies from 179 (*SlADH17*) to 511 (*SlADH34*), but most members have between 300 and 400 amino acids, except for *SlADH17*, *SlADH15*, and *SlADH34*. The genome length of *SlADH* family members is generally large, but the corresponding number of encoded amino acids is not particularly large, indicating that introns account for a large proportion of the genetic structure of the gene family. The molecular weight of SlADH protein ranges from 19.16 kDa (SlADH17) to 56.49 kDa (SlADH34), and the theoretical pI ranges from 6.01 (SlADH30) to 9.84 (SlADH8). Furthermore, out of the total, 21 SlADH proteins are anticipated to be alkaline (with a pI > 7.0), while 15 are predicted to be acidic (pI < 7.0).

**TABLE 1:** The basic information of *SlADH* genes.

Gene name	Gene ID	Chr	Start	End	Aalen	MolWt	pI
*SlADH1*	Solyc01g006510.3.1	ch01	1100167	1104497	356	38126.33	6.24
*SlADH2*	Solyc01g107590.3.1	ch01	87399828	87403587	358	38976.03	6.18
*SlADH3*	Solyc02g030480.4.1	ch02	24786495	24790582	361	39526.46	7.01
*SlADH4*	Solyc02g069250.4.1	ch02	37203429	37205538	377	41455.83	8.47
*SlADH5*	Solyc02g078940.3.1	ch02	41536132	41540597	326	34669.05	7.27
*SlADH6*	Solyc03g044200.3.1	ch03	7742938	7746890	384	41832.66	7.12
*SlADH7*	Solyc03g078440.3.1	ch03	45470488	45473348	392	42796.67	8.38
*SlADH8*	Solyc03g095360.3.1	ch03	50989703	51003940	349	36991.17	9.84
*SlADH9*	Solyc04g064710.3.1	ch04	55204700	55207798	380	41151.26	6.88
*SlADH10*	Solyc04g074530.3.1	ch04	58480276	58485032	395	42697.45	8.01
*SlADH11*	Solyc04g074535.1.1	ch04	58485264	58489652	385	41499.78	6.36
*SlADH12*	Solyc04g082170.3.1	ch04	63916846	63920140	390	42374.18	6.88
*SlADH13*	Solyc04g082180.4.1	ch04	63920302	63924270	391	42382.05	6.6
*SlADH14*	Solyc05g005480.2.1	ch05	398261	401636	389	41001.13	8.6
*SlADH15*	Solyc05g056540.4.1	ch05	65198800	65203831	433	46103.96	7.81
*SlADH16*	Solyc06g034120.4.1	ch06	21440315	21447770	384	41137.49	8.94
*SlADH17*	Solyc06g035680.3.1	ch06	22468209	22469101	179	19168.87	6.89
*SlADH18*	Solyc06g059740.4.1	ch06	35287450	35289927	357	38828.04	7.26
*SlADH19*	Solyc06g072160.3.1	ch06	42144939	42149927	395	42184.61	6.05
*SlADH20*	Solyc08g014360.2.1	ch08	4139699	4145882	381	41816.43	8.53
*SlADH21*	Solyc08g083280.3.1	ch08	63931227	63933681	382	41449.93	6.66
*SlADH22*	Solyc09g059030.4.1	ch09	49092687	49096229	330	34687.21	8.75
*SlADH23*	Solyc09g059040.3.1	ch09	49131894	49135604	330	34471.85	9.59
*SlADH24*	Solyc09g064370.4.1	ch09	57657017	57661659	313	33628.83	7.87
*SlADH25*	Solyc11g010960.2.1	ch11	4037649	4041848	360	39214.5	6.89
*SlADH26*	Solyc11g010980.2.1	ch11	4046446	4050519	360	38994.14	6.98
*SlADH27*	Solyc11g010990.3.1	ch11	4057113	4060036	360	38992.13	6.93
*SlADH28*	Solyc11g011330.2.1	ch11	4405726	4407631	357	38776.52	6.29
*SlADH29*	Solyc11g011340.2.1	ch11	4418827	4421802	360	39373.87	7.96
*SlADH30*	Solyc11g071290.2.1	ch11	52889224	52895870	377	41179.67	6.01
*SlADH31*	Solyc11g072640.3.1	ch11	53926630	53936354	379	41295.75	9.38
*SlADH32*	Solyc11g072650.3.1	ch11	53938063	53941127	357	39323.38	7.02
*SlADH33*	Solyc12g014050.2.1	ch12	4900730	4907725	397	42881.34	8.46
*SlADH34*	Solyc12g055820.3.1	ch12	61410511	61417343	511	56495.98	7.56
*SlADH35*	Solyc12g094500.3.1	ch12	64212297	64216095	390	42060.55	6.17

Chr, chromosome number; Aalen, amino acid length; MolWt, molecular weight; pI, isoelectric point.

**FIGURE 1:**
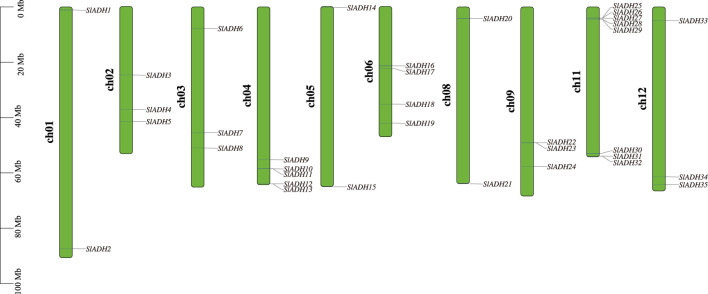
Chromosomal distributions of *SlADH* genes. Chromosome number is indicated.

A total of 35 *SlADH* genes were identified on 12 tomato chromosomes, and their distribution on the chromosomes was uneven. All chromosomes, except for the 7th and 10th, contain the SlADH gene, as depicted in Figure 1. Among the 10 chromosomes, *SlADH* family members are mainly distributed on chromosomes 4 and 11. Chromosome 11 contains the most *SlADH* family members, 8 in total, and the positions of these members on chromosome 11 are very close to each end. In general, the distribution of members of this family on chromosomes is found that most members of this family are distributed at both ends of chromosomes, only three members exist in the middle of chromosomes 2 and 6.

### 3.2 Phylogenetic relationships among *SlADH* genes

**FIGURE 2:**
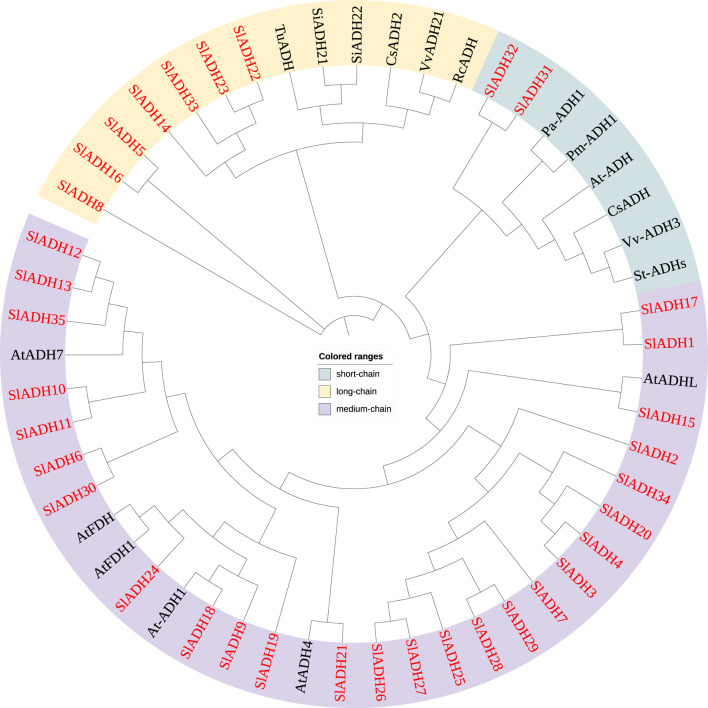
Phylogenetic tree of ADH proteins.

In order to understand the possible functional differences among individual members of tomato *SlADH* gene superfamily, the sequences of a set of *ADH* family members from other plant species, including *Solanum lycopersicum* (Sl), *Triticum urartu* (Tu), *Setaria italica* (Si), *Cucumis sativus* (Cs), *Vitis vinifera* (Vv), *Rosa chinensis* (Rc), *Prunus armeniaca* (Pa), *Prunus mume* (Pm), and *A*. *thaliana* (At), were analyzed using a Neighbor-joining (NJ) phylogenetic tree. As shown in Figure 2, the phylogenetic tree divided these *ADHs* into three clades: short-chain *ADHs*, medium-chain *ADHs* and long-chain *ADHs*. Among the 35 *SlADH* family members of tomato, most belong to the medium-chain *ADH* branch (26 members), while the short-chain *ADH* branch contains the least *SlADH* family members, only two. It is not difficult to see from the evolutionary relationship that members of the *SlADH* family are closer to *A. thaliana* than other species, but far from masson pine, rose and other woody plants, so they contain the least members in the short-chain *ADH* branch.

### 3.3 Conserved motifs and gene structure analyses of *SlADH* genes

**FIGURE 3:**
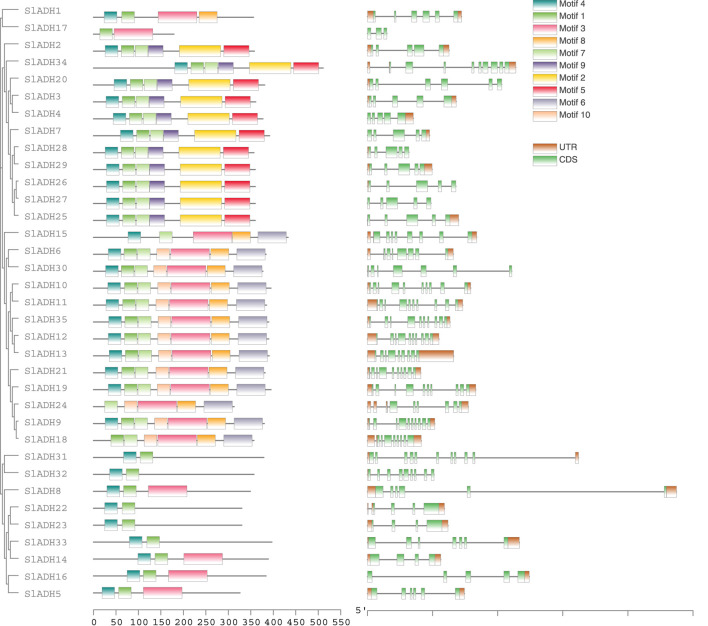
Conserved motifs, conserved domain and gene structure of tomato ADH proteins.

We used MEME tool to analyze the conserved motif of SlADH protein. A total of 10 distinct motifs were detected in all 35 SlADH proteins. While some members showcased distinct motif variations, underscoring the diversity of SlADH proteins, a majority exhibited highly similar motifs. Meanwhile, through the analysis of conserved motifs, we speculated that the proteins expressed by SlADH family members might play a conservative and single function. Motif 4 is present in all proteins except SlADH18, suggesting that this motif may be a conserved structure of the SlADH family. Then, in order to study the exon-intron structure of SlADH gene, we used the gene structure display server program to visually analyze the SlADH gene genome and coding sequence. Of the 35 *SlADH* genes, all members contain introns. In addition, all except *SlADH17* and *SlADH27* contain UTRs (Figure 3). *SlADH20*, *SlADH26*, *SlADH28*, *SlADH30*, and *SlADH32* contain only 3′ UTR, while *SlADH4*, *SlADH7*, and *SlADH15* contain only 5′ UTR.

### 3.4 Duplication analysis of tomato *SlADH* genes

**FIGURE 4:**
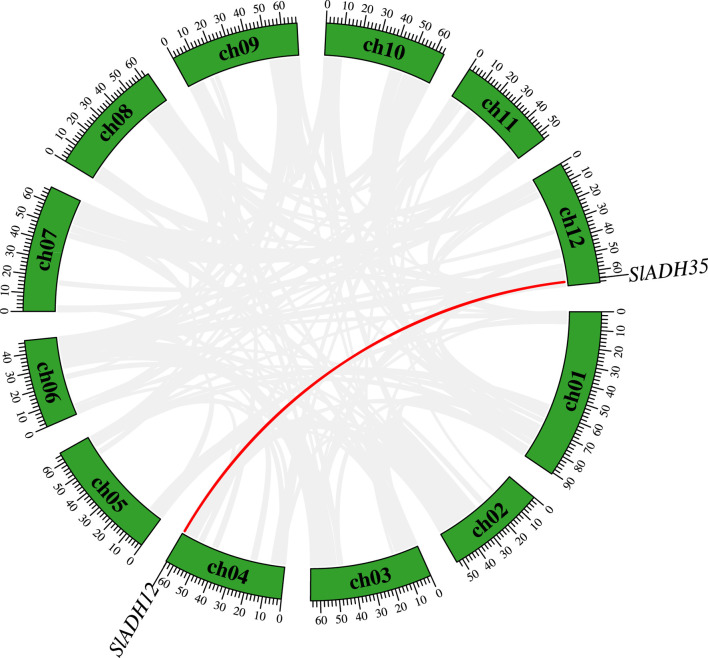
The duplication genes in *SlADH* gene family. Segmental duplication gene pairs inked by red line.

Analyzing gene duplication is pivotal, as it often unravels insights into evolutionary processes and the functional diversification or redundancy of genes, potentially driving the adaptability and evolution of the species. Duplication events among *SlADH* members were analyzed (Figure 4). There was only one duplicate gene pair in 35 *SlADH* genes (*SlADH12/SlADH35*), which reflected the functional specificity and diversity of the members of this family.

### 3.5 Promoter cis-element analysis of *SlADH* genes

**FIGURE 5:**
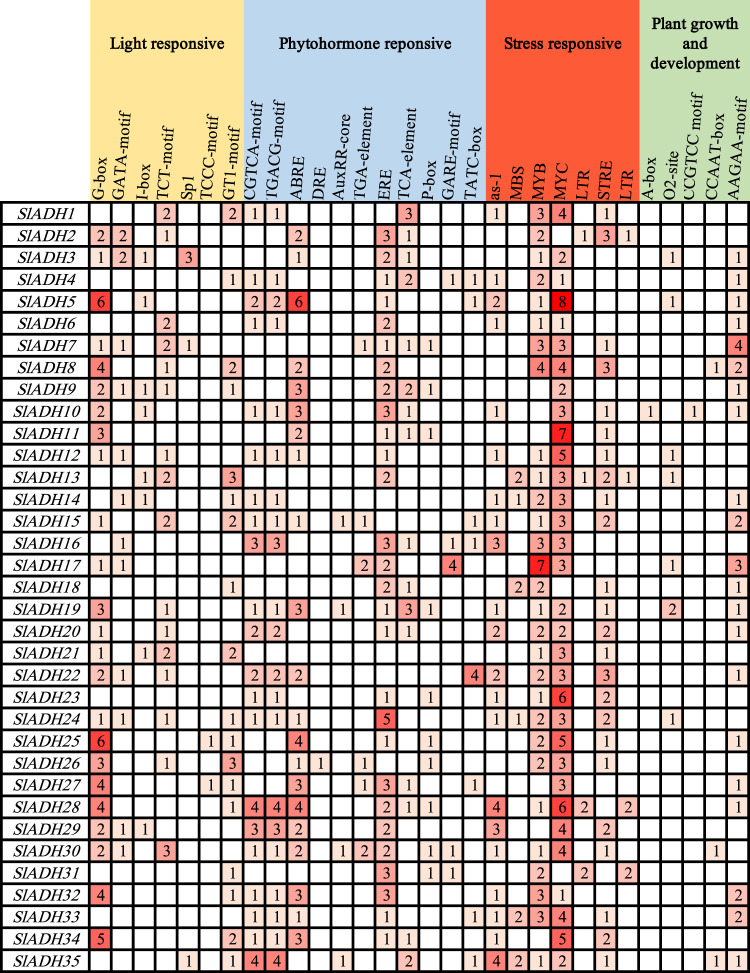
Cis-acting element analysis in *SlADH* promoter regions.

To reveal the diversity of cis-elements in the promoter region of the *SlADH* gene, we selected the 1,500 bp sequence upstream of the *SlADH* gene and submitted it to the PlantCARE database for promoter cis-element analysis (Figure 5). We then visualized the results, and found that there were seven elements related to light responsive and seven elements related to stress response. The largest number of elements related to phytohormone reponsive were 11. There were only five elements related to plant growth and development. In these homeopathic elements, MYC is widely distributed throughout the *SlADH* family species, with only three members lacking it. As a key component of plant response to various stresses, such a large amount of MYC also reflects the key role of *SlADH* family in plant.

### 3.6 Synteny analysis of *ADH* genes between tomato and arabidopsis, potato, maize

**FIGURE 6:**
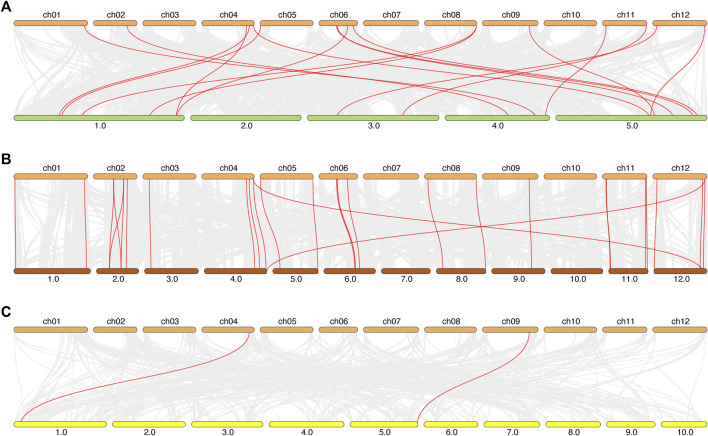
The synteny relationship between tomato and Ara, rice and potato. **(A)**: tomato and Ara, **(B)**: tomato and potato, **(C)**: tomato and maize. All the synteny relationship *ADH* genes between different species were linked by red, the collinear blocks within the tomato and other plant genomes were linked by gray lines in the background.

To support the presented information about the evolutionary process in tomato, we investigated the syntenic relationships of the *ADH* gene pairs in different species (Figure 6). We selected the model plant *A. thaliana*, the Solanaceae crop potato, and the monocotyledon crop maize to explore inter-species synteny relationship *ADH* genes. The results showed that there were 17 pairs of *ADH* genes between tomato and *A. thaliana*, 28 pairs of *ADH* genes between tomato and potato, and only two pairs of *ADH* genes between maize and tomato.

### 3.7 Expression pattern analysis of tomato *SlADH* gene family members under stress

**FIGURE 7:**
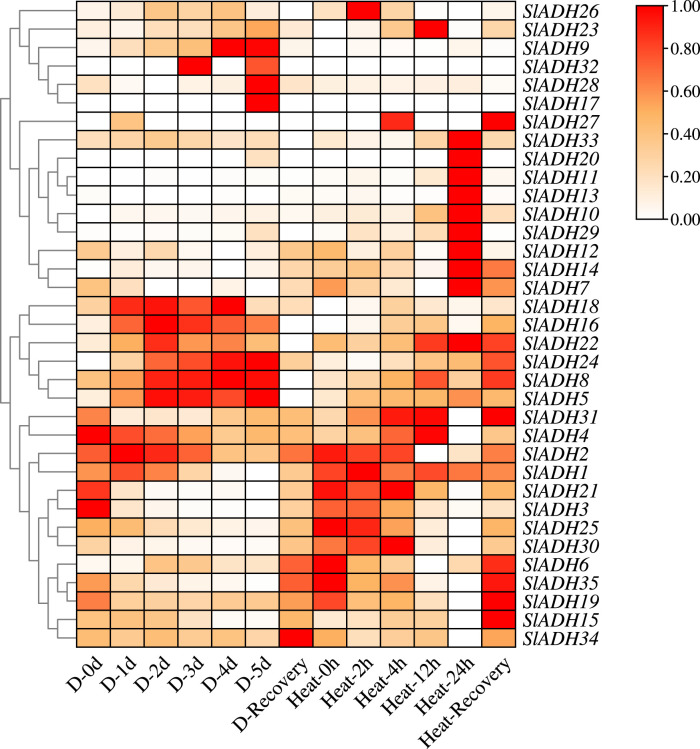
Relative expression levels of *SlADH* genes in transcriptome.

From the transcriptome data, following drought and heat stress, SlADH family members predominantly exhibited two patterns: first increase, then decrease and gradually increase (Figure 7), and only a few members did not respond strongly. In drought stress, most members reached the highest expression level of *SlADH* gene on the third day after drought stress, whereas, during heat stress, the highest expression levels of SlADH family members were observed at 12 and 24 h. For both stresses, *SlADH* family members showed an extremely significant response trend in the recovery treatment after stress. The level of expression increased dramatically. This suggests that the SlADH family members play a pivotal role not just in stress resistance but also in reestablishing plant homeostasis following the stress.

### 3.8 qRT-PCR analysis of *SlADH* gene under abiotic and ABA treatments

**FIGURES 8:**
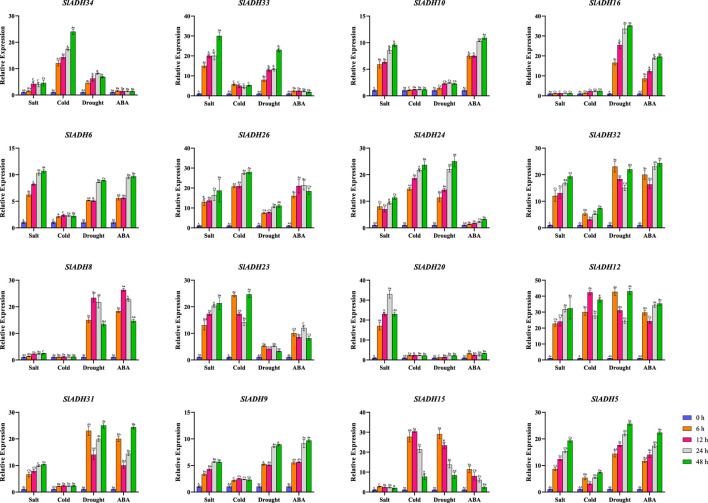
Expression levels of 16 selected *SlADH* genes in response to salt, cold, drought and ABA stress.

Subsequently, tomato plants were treated with salt stress (NaCl), cold stress, drought stress (PEG) and ABA treatment, and a total of 16 genes from different subbranches of the phylogenetic tree were selected for response analysis (Figure 8). All 16 selected family members exhibited responses to at least one of the stresses or treatment. Of the 16 selected gene family members, 13 *SlADH* genes responded to salt stress (except *SlADH16*, *SlADH8*, *SlADH15*), 11 *SlADH* genes responded to cold stress(except *SlADH10*, *SlADH16*, *SlADH8*, *SlADH20*, *SlADH31*), 11 *SlADH* genes responded to drought stress(except *SlADH20*), and 12 *SlADH* genes responded to ABA treatment(except *SlADH34*, *SlADH33*, *SlADH24*, *SlADH20*). Among them, *SlADH6*, *SlADH26*, *SlADH24*, *SlADH32*, *SlADH12*, *SlADH9*, and *SlADH5* all responded to the four kinds of stress. Except for *SlADH12*, which showed no obvious trend, the expression levels of the other genes all showed a trend of gradual increase after stress. In addition, *SlADH15* showed obvious response to cold stress, drought stress and ABA treatment except salt stress, and the expression level decreased gradually after a significant increase. However, *SlADH20* was only responsive to salt stress, showing a trend of first increasing and then decreasing. *SlADH16* and *SlADH8* showed a good response to drought stress and ABA treatment, but no significant response to the other two stresses. These results suggest that the SlADH family members are responsive to a majority of stresses or treatments, underscoring their potential significance in plant reactions to diverse abiotic challenges. Concurrently, individual family members exhibit varied response patterns to distinct stresses, highlighting the functional diversity and specificity of these genes.

## 4 Discussion

In recent years, extreme weather has occurred frequently around the world. Drought, waterlogging, salinity and freezing damage are all environmental factors that threaten plant growth. Adverse environmental conditions have a negative impact on agricultural production and reduce both the qualitative and quantitative yields of crops. Climate change is expected to pose serious challenges to agricultural production worldwide by causing rising temperatures and water shortages. Exploring the molecular responses of plants to abiotic stresses and using this knowledge to develop crop plants that can adapt to these adverse environmental conditions and maintain high yields under these adverse environmental conditions has been a major goal of molecular breeders (Xiong et al., 2002; Umezawa et al., 2006; Seki et al., 2007; Shinozaki and Yamaguchi-Shinozaki, 2007; Hirayama and Shinozaki, 2010; Zhu, 2016; Fontemaggi, 2023). Despite concerted efforts and significant findings, this challenge persists, highlighting its complexity and the need to develop new methods to mitigate the damage caused by environmental stress. As a result, identifying robust resistance genes in plants has emerged as a primary solution.

Over the past few decades, several studies have characterized *ADH* genes, and this work has greatly increased our understanding of plant responses to stress in many species. Many studies of *ADH* gene expression have linked it to both biotic and abiotic stresses. However, until now, the *ADH* gene family has not been reported in tomato, which has hindered the study on the function of tomato *ADH* gene to a certain extent. On this basis, we systematically analyzed the *ADH* gene family of tomato using bioinformatics. In this study, 35 *ADH* genes were identified from tomato, which had more *ADH* family members than maize (seven members) and grape (ten members) (Su et al., 2020). The members of this gene family are characterized by long coding sequence but few amino acids, and most members of this family have a gene structure with many introns. At the same time, the molecular weight of the total family members proteins were similar, and most of the proteins were alkaline proteins, and the overall distribution was relatively neutral (pI = 7). This may indicate that the proteins in this family have a more conservative function. Then, through duplication analysis of the tomato *SlADH* family members, we found that there was only one gene pair (*SlADH12*/*SlADH35*) among the 35 members, which indicated the functional diversity of the family members. In motif analysis, it is found that there are 10 motifs in this family, which are evenly distributed in different members of the family, forming a variety of different forms in the way of combination. We speculate that it is this relatively uniform combination distribution that causes the specificity and diversity among the members of this family, and they likely have more specific functions. The results of conserved motifs, gene characteristics and gene structure analysis of *ADH* family genes supported the classification results of phylogenetic analysis. We found that the signature of the *ADH* gene was similar within each subfamily and varied between different subfamilies.

In addition, four types of cis-acting elements were identified through the analysis of *SlADH* gene family, which were related to light response, phytohormone reponsive, stress response and plant growth and development. Among these elements, the types of elements related to phytohormone reponsive were the largest, but the total number of elements related to stress was the largest. Of these elements, MYC is the most numerous, with only three members without MYC element. MYC is one of the members of bHLH transcription factor superfamily, and MYC has been widely reported. MYC plays a very important role in plant growth and development, especially in stress resistance (Abe et al., 1997; Perez-Alonso et al., 2021). In addition, most family members also include MYB element and LTR element. Research has shown that, overexpression of *MYB12* and *MYB75* in transgenic plants significantly increased the accumulation of flavonoids with strong antioxidant activity, thus resulting in enhanced tolerance to abiotic stresses such as drought and oxidative stresses (Nakabayashi et al., 2014; Wang et al., 2016). And LTR elements may be potential molecular targets for temperature domestication (Wu et al., 2019). Meanwhile, stress-related elements such as *MBS* and *STRE* also make up a large portion. The presence of such a large number of stress-related response elements in the gene structure further demonstrated the key role of *SlADH* gene family in tomato resistance process.

Subsequently, we analyzed the expression level of tomato *SlADH* gene family after heat stress and drought stress by transcriptome data, and selected 16 genes for qRT-PCR. The results showed that the q-PCR trend of the selected genes was consistent with that of the transcriptome. This verified the authenticity and reliability of transcriptomic data. In the entire transcriptome, most members of the *SlADH* gene family showed a response to heat stress and showed an increasing tendency after the plants were treated with heat stress. Then, we subjected tomato to salt stress (NaCl), cold stress, drought stress (PEG) and ABA treatment, and selected 16 *SlADH* family members for expression level detection. Through the above experiments, we once again verified that *SlADH* gene family has a certain degree of response to a variety of abiotic stresses, indicating that *SlADH* gene family members play a key role in the process of tomato abiotic stress. Studies have shown that there are three *ADH* genes in Pygmy cattle, and two of them are induced by hypoxia (Garabagi et al., 2005). Similarly, *ADHA* of two *ADH* genes in cotton is induced by waterlogging (Estrada et al., 2022; Pan et al., 2023), indicating that *ADH* gene family plays a vital role in plant resistance to waterlogging. Low temperature can cause lipid peroxidation of plant cell membrane, and even death of some plant cells sensitive to low temperature (Kidokoro et al., 2022). Noguchi et al. found that low temperature stress (5°C, 7.5°C, 10°C) can significantly improve the activities of *ADH* and *PDC* in rice seedlings, and enhance the ethanol fermentation pathway. Owing to its unique gene function, ADH not only holds significance in diverse biotic and abiotic stress responses but also assumes a pivotal role across various domains. *ADH*, a zinc-binding enzyme, is a key enzyme in the metabolic pathway of fatty acids, which can reversibly convert aldehydes into corresponding alcohols and may play an important role in the synthesis of volatile esters. So the gene also plays a crucial role in regulating fruit flavor. In *ADH*, not only the medium-chain *ADH* gene is associated with the production of aromatic volatiles (Singh et al., 2010; Bajpai et al., 2018), the short chain *ADH* may contribute to the biosynthesis of plant aroma (Moummou et al., 2012). Other studies have shown that *ADH* genes are expressed in a developmentally regulated manner, especially during fruit ripening (Manriquez et al., 2006). *LeADH2* is expressed in tomato fruits and its abundance increases during ripening, especially in late ripening (Longhurst et al., 1994). Similarly, *VvADH* expression is noted during the late ripening phase of grapes (Tesniere and Verries, 2000). A similar pattern was observed in Nanguo pear, especially in varieties with high expression of *ADH6* and *ADH7*. The augmented transcriptional accumulation of *ADH6* in ripe Nanguo Pear fruit correlates with heightened ADH activity, akin to the scenario with *VvADH1* in grape (Tesniere and Verries, 2000). Qin et al. found that according to the transcription mode of *ADH*, *ADH* activity and hexyl ester synthesis in Dangshansu pear and Nanguo pear fruits are related to the transcription of *ADH6*, indicating that *ADH* plays a decisive role in the volatiles of pear fruits (Zeng et al., 2020). Both stress resistance and fruit quality will have a direct impact on the economic value of tomato and directly affect the income of farmers. Therefore, through bioinformatics analysis and omics data analysis of *ADH* gene, this paper reveals the important role of *ADH* gene on tomato stress resistance and fruit quality to some extent. It can provide a strong reference for further exploration of its function.

## 5 Conclusion

In conclusion, this study identified *SlADH* gene family members in tomato for the first time. Bioinformatics analysis and qRT-PCR analysis under abiotic stress revealed that tomato *SlADH* gene family members may play an important role in tomato growth and development and stress resistance. This investigation underscores the pivotal significance of the *SlADH* gene in tomato and establishes a foundational platform for subsequent inquiries into the broader role of the *SlADH* gene family in tomato breeding and augmenting resistance.

## Data Availability

The original contributions presented in the study are included in the article/Supplementary Material, further inquiries can be directed to the corresponding author.
