# Supplementary material for: Genome-wide identification and characterization of ADH gene family and the expression under different abiotic stresses in tomato (Solanum lycopersicum L.)
Source: Front Genet. 2023 Sep 1;14:1186192. doi: 10.3389/fgene.2023.1186192 (PMC10506264; doi:10.3389/fgene.2023.1186192)
Supplement: Supplementary file 1 [file Table1.DOCX]

Table S1. The sequences of primers for qRT-PCR analysis

| **Primer name** | **Primer sequences** |
| --- | --- |
| SlADH34-RT-F | 5’-ATGTGTTGCCCTGGTTGTGA-3’ |
| SlADH34-RT-R | 5’-GTGGTGGATTTGGGTATGGC-3’ |
| SlADH33-RT-F | 5’-ATGCGATCAGGCTATGGACG-3’ |
| SlADH33-RT-R | 5’-AAGTGATCCCGGCTTTGGAG-3’ |
| SlADH10-RT-F | 5’-ATAGAGATGCACGGCACACC-3’ |
| SlADH10-RT-R | 5’-AACAGGATTATTTGCCCATCCA-3’ |
| SlADH16-RT-F | 5’-AAGTTGTGCCTGTTCCTCCC-3’ |
| SlADH16-RT-R | 5’-GACTGTAGCACCAAGGGCAT-3’ |
| SlADH6-RT-F | 5’-TGCTCTTTGCCAGTTTGTGC-3’ |
| SlADH6-RT-R | 5’-CCTGACTTGCAATTTGGGCA-3’ |
| SlADH26-RT-F | 5’-AAGCCACTTGAGTTGCCAGT-3’ |
| SlADH26-RT-R | 5’-TCGAAAGACGTCCCATAGCAA-3’ |
| SlADH24-RT-F | 5’-AGGCTGCGGGGTACGATA-3’ |
| SlADH24-RT-R | 5’-TCGACTCTTACGGTCGTTCA-3’ |
| SlADH32-RT-F | 5’-CCCGTAGACACCGTCATCAG-3’ |
| SlADH32-RT-R | 5’-GAGTCTTCACAGCAGAGCCA-3’ |
| SlADH8-RT-F | 5’-GAAGTACCTGTCGTCCCAGC-3’ |
| SlADH8-RT-R | 5’-AAGCAAGATGGGCCTCTGTC-3’ |
| SlADH23-RT-F | 5’-ATTCACTGCACGACAGGCAT-3’ |
| SlADH23-RT-R | 5’-TTCCCTTCCTTCACAAGCCC-3’ |
| SlADH20-RT-F | 5’-CTGCTCAAGACTCCTCTGGC-3’ |
| SlADH20-RT-R | 5’-TGACACATTGCTTCCCACCT-3’ |
| SlADH12-RT-F | 5’-GGCTATGTGGAGCTACCAGG-3’ |
| SlADH12-RT-R | 5’-GCTTCTTGCACAAGTGTTGCT-3’ |
| SlADH31-RT-F | 5’-GCTGCCTCAAGTTCCAAAGC-3’ |
| SlADH31-RT-R | 5’-AGTGCAAACTGGTTCCTCGT-3’ |
| SlADH9-RT-F | 5’-TCAGCAATGCCGTCACTGTA-3’ |
| SlADH9-RT-R | 5’-TGGTGACGCAACCAGAATGA-3’ |
| SlADH15-RT-F | 5’-TCCATTCGCTAGCCCTTGTG-3’ |
| SlADH15-RT-R | 5’-GTTCCCTTCGCTCGGTTGTA-3’ |
| SlADH5-RT-F | 5’-CCCCTAAAGGCGACAGTGAA-3’ |
| SlADH5-RT-R | 5’-CAGGAAAGCTAGCCGCATCT-3’ |
